# Increased γ-Aminobutyric Acid Content of Germinated Brown Rice Produced in Membrane Reactor

**DOI:** 10.17113/ftb.59.03.21.6846

**Published:** 2021-09

**Authors:** Azis Boing Sitanggang, Michael Joshua, Hadi Munarko, Feri Kusnandar, Slamet Budijanto

**Affiliations:** Department of Food Science and Technology, IPB University, Campus IPB Darmaga, 16680 Bogor, Indonesia

**Keywords:** germinated brown rice, IPB 3S rice variety, membrane reactor, γ-aminobutyric acid, γ-oryzanol

## Abstract

**Research background:**

Rice germination is a natural approach to enhance the physical and functional properties of brown rice. Thus, the aim of this study is to investigate the influence of different germination methods on functional properties of germinated brown rice and evaluate the process feasibility.

**Experimental approach:**

Brown rice of IPB 3S variety was germinated with three different methods: (*i*) complete soaking without water replacement, (*ii*) complete soaking with water replacement every six hours, and (*iii*) complete soaking with continuous washing in the developed membrane-facilitated soaking reactor.

**Results and conclusions:**

The application of the membrane reactor for producing germinated brown rice maintained the pH of the soaking solution relatively constant (*i.e.* 6.8-7.0). This indicated the circumvention of natural fermentation during brown rice germination. Moreover, the mass fraction of γ-aminobutyric acid in germinated brown rice produced in the membrane reactor was about 4.5-fold higher (169.2 mg/100 mg) than in ungerminated brown rice (36.82 mg/100 mg), and also higher than that of the other two soaking methods. The γ-oryzanol mass fractions and the antioxidant capacity expressed as ascorbic acid equivalents of germinated brown rice obtained with the three soaking methods varied from 32 to 38 mg/100 mg and 18 to 28 mg/100 g, respectively. Within this study, germination could also slightly reduce the transition temperatures of germinated brown rice starch gelatinization (*t*_o_=73-74 °C, *t*_p_=76-77 °C and *t*_c_=~80 °C, where *t*_o_, *t*_p_ and *t*_c_ are onset, peak and conclusion (final) temperatures). In conclusion, the production of germinated brown rice in the membrane reactor could enhance its γ-aminobutyric acid mass fraction and reduce wastewater production and is therefore considered more feasible.

**Novelty and scientific contribution:**

This study demonstrates the feasibility of germinated brown rice production using a membrane-facilitated soaking reactor with enhancement of bioactive compound content, especially γ-aminobutyric acid, and minimised wastewater production.

## INTRODUCTION

Rice (*Oryza sativa* L.), as one of the main sources of carbohydrate, is considered staple food for more than one third of the world's inhabitants (*1*). Rice is widely produced, especially in Asian countries, with Indonesia occupying the third position after China and India (*2*). The high consumption of rice in Asian countries is indicated by the level of rice-based calorie fulfilment, which may reach up to 70%.

As a product of the dehusking, brown rice still has a layer of the epidermis called rice bran. It is rich in health-promoting components, such as γ-oryzanol, tocopherols, tocotrienols, dietary fibre and plant sterols. Although brown rice has advantages over white (or polished) rice in terms of its nutritional contents, its consumption is still less preferred (*3*). Besides, the awareness about its nutritional and functional properties is poor; several barriers for higher brown rice consumption are inferior cooking properties, lower sensory acceptance and limited shelf life (*4*). The longer cooking time and low digestibility in relation to the hard texture of brown rice are perceived as low-grade characteristics. Additionally, due to the presence of phenolic contents and fibre in the rice bran, its sensory attributes are always associated with an astringent taste and nutty flavour (*3*).

Amongst various approaches introduced to increase brown rice acceptance, germination has been considered as the most suitable method to especially ameliorate brown rice sensory attributes, nutrient contents and functional compounds (*5*, *6*). Significant biochemical changes during germination lead to the increase in free amino acids, reducing sugars (due to the action of amylolytic enzymes), γ-aminobutyric acid (GABA) and soluble fibre content in germinated brown rice (*7*).

In general, two conventional approaches are adopted to produce germinated brown rice, complete soaking of brown rice until 0.5-1.0 mm sprouts appear and a combination of soaking (at 25-45 °C for 12-24 h) and incubation under humid conditions (relative humidity of 65% for up to 48 h) (*8*). The optimisation of germinated brown rice production is commonly reported based on GABA content (*9*). Optimum pH of the soaking water, the combination of soaking or incubation time, and temperature were previously investigated (*8*, *10*). Additives such as glutamic acid or gibberellin are sometimes added into soaking water to enhance GABA content (*11*). In addition to this, Chen *et al.* (*9*) reported the application of a low-pressure plasma to produce germinated brown rice with high GABA content. With this treatment, the antioxidant activity of the rice also increased.

From the perspective of scale-up and the ease of adoption to industrial scale, such conventional approaches of producing germinated brown rice are considered feasible. However, either taking the first or second conventional approach, a large amount of soaking wastewater may entail at the end of production. During soaking, the replacement of water has to be done every three to six hours to avoid natural bacterial fermentation to occur (*12*). Although brown rice is pretreated using electrolysed oxidising water (to decontaminate a broad spectrum of microbes), the water still needs to be replaced every 12 h (*13*). Moreover, at least one operator is needed to perform this procedure. To mitigate the drawbacks of the conventional approaches (*i.e.* to reduce wastewater and manpower), within this work, we designed and developed a membrane reactor to produce germinated brown rice. As a proof-of-concept, the germination of rice variety IPB 3S was performed in this reactor. Besides utilising the membrane reactor, the rice was also germinated through complete soaking without water replacement and complete soaking with water replacement every six hours. The impact of each method on functional properties of germinated brown rice and process feasibility were thus evaluated.

## MATERIALS AND METHODS

### Materials

Unhulled rice (IPB 3S variety) was obtained from PT Bogor Life Science and Technology (BLST, Bogor, Indonesia). Chemicals such as ethanol, methanol, 2-propanol, phenol, anhydrous sodium tetraborate, boric acid, l-ascorbic acid, γ-oryzanol, γ-aminobutyric acid (GABA) and sodium hypochlorite were purchased from Merck KGaA (Darmstadt, Germany). The 2,2 diphenyl-1-picrylhydrazyl (DPPH) was purchased from Shanghai Chaining Chemicals Co., Ltd. (Shanghai, PR China).

### Design of membrane reactor

**Fig. 1:**
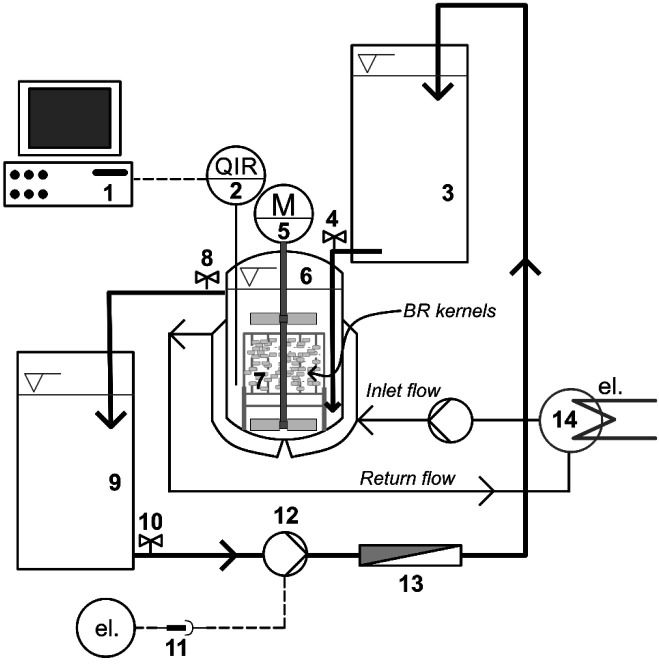
Design of the membrane reactor for producing germinated brown rice: 1=PC, 2=pH and temperature sensor, 3=container 1: clean water tank, 4=valve 1, 5=stirrer, 6=container 2: double jacketed reactor, 7=soaking basket, 8=valve 2, 9=container 3: spent water tank, 10=valve 3, 11=digital timer for electricity connection, 12=liquid pump, 13=microfiltration (MF) membrane unit, and 14=water bath system

The design of the developed membrane reactor is shown in Fig. 1 (*14*). The system consisted of three containers made of poly(methyl methacrylate): container 1 for clean water reservoir, *d*=0.24 m, *h*=0.60 m (no. 3 on the diagram), container 2 was a double jacketed reactor, *d*=0.21 m, *h*=0.36 m (no. 6 on the diagram) and container 3 was spent water reservoir, *d*=0.24 m, *h*=0.45 m (no. 9 on the diagram). At the bottom of containers 1 and 3, a manual valve was mounted and kept open throughout the soaking. For the reactor itself (container 2), at 70% of its height, a valve was also mounted to control the reactor volume constantly through the overflow. It is worth noting that these three containers were placed at different heights, to facilitate water flow from one container to another. A soaking basket, *d*=0.17 m, *h*=0.21 m was placed inside the reactor (no. 7). The agitation (double blade stirrer, *D*/*d* ratio 1.167) was performed with IKA RW 20 Digital stirrer (IKA-Werke GmbH & Co. KG, Staufen, Germany) (no. 5). The stirrer shaft protruded through the soaking basket adjacent to the reactor base. The reactor was also equipped with WTW 3310 pH meter (Xylem Analytics GmbH, Weilheim, Germany) (no. 2), and the serial interface was connected to a PC (no. 1). The reactor was double jacketed with a water bath system THH-2 (Lab Tehnik, Jakarta, Indonesia) connected to it (no. 14). The spent water was discharged through valve 3 (no. 10), filtered using microfiltration membrane (Nanotec® Filter 0.1 μm) (no. 13), and conveyed back to the upper container (no. 3) utilizing a P-WH75 IPX4 pump (Sanyo, Jakarta, Indonesia) (no. 12).

### pH and temperature measurement

The real-time monitoring and recording of pH and temperature were performed during soaking. For data acquisition, a program was developed using Laboratory Virtual Instrumentation Engineering Workbench (LabVIEW; National Instruments, Austin, TX, USA). The transmission setting of pH meter had a baud rate of 4800 bit/s, and stop bit/s of 8 and 2, respectively. Within the acquisition program, the VISA function was used to establish communication between the pH meter and the PC (*15*). The transferred string regularly contained 128 characters, where the pH and temperature values were encrypted. Within the acquisition program, ’match pattern’ and ’scan from string’ functions were used for extracting the relevant information from the transferred string. The information was then documented in the text as a logical volume management (LVM) file every second (*16*).

### Preparation of brown rice

Brown rice was obtained by dehulling (model THU35B; SATAKE, Hiroshima, Japan) the IPB 3S variety. Rice grader OSK 10291R (Ogawa Seiki. Co., Ltd., Okubo Shinjuku-ku, Japan) was used to separate a mixture of whole kernels and head-rice kernels from broken kernels. Furthermore, through manual sorting, only whole kernels were used for the production of germinated brown rice. The shape of the whole kernel was slender with an average length and width of (7.25±0.31) and (2.14±0.08) mm, respectively.

### Production of germinated brown rice

Brown rice was soaked in distilled water (pH=6.8±0.2, *t*=(28±2) °C) with a brown rice-to-water ratio 1:10. Three soaking methods were investigated in this study: soaking without clean water replacement, soaking with manual water replacement every six hours, and continuous operation of the membrane reactor. For the first two methods, the double jacketed reactor (no. 6 in Fig. 1) was also used without agitation. For soaking with membrane reactor, the water from container 1 (*V*=~27.14 L) was continuously fed to the reactor through valve 1. The excessive amount of water in the reactor flowed to container 3 through valve 3. The spent water in container 3 was filtered and recycled into container 1. The characterisation of fouling on the microfiltration membrane module during the third soaking method was not part of the interest of this study. Additionally, the agitation was introduced in the reactor throughout the soaking at 110 rpm. The samples were withdrawn at 24, 48, 72, 96 and 120 h. Germinated brown rice kernels were photographed using Canon 500D (Canon Inc., Tokyo, Japan) to compare their morphologies based on the treatments. Furthermore, kernels were then dried using a tray dryer AM-TD 24 (Aneka Mesin, Jakarta, Indonesia) at 50 °C for 12 h. Moreover, the dried samples were comminuted (NM 8300 Nima, Oda, Japan) to pass through 0.25 mm (60 Tyler mesh) sieve, and stored at 4 °C before analyses.

### Determination of GABA mass fraction

One gram of (germinated) brown rice flour was poured into 5 mL distilled water in a 15-mL benchtop shaker and shaken (New Brunswick™ Innova® 2300, Eppendorf AG, Wesseling-Berzdorf, Germany) at 200 rpm and 30 °C for 60 min. Following this, the slurry was centrifuged (centrifuge 5810 R; Eppendorf AG) at 1008×*g* and 30 °C for 60 min. The supernatant was then filtered using a nylon membrane *d*(pore)=0.45 μm (Axiva Sichem Biotec., Delhi, India).

GABA mass fraction was determined based on ω-amino acid analytical procedures (*1*). A volume of 0.5 mL of sample, 0.2 mL of 0.2 M borate buffer and 1.0 mL of 6% (*m*/*V*) phenol reagent were mixed thoroughly. A volume of 0.4 mL of 9% (*m*/*V*) sodium hypochlorite reagent was added to the mixture. The tube was then vortexed, placed in boiling water for 10 min, and immediately cooled down in ice for 20 min. The absorbance was measured at 645 nm using UV-Vis spectrophotometer Genesys™ 150 (Thermo Fisher Scientific, Waltham, MA, USA). A blank was made by replacing the sample with distilled water. The mass fraction of GABA was calculated based on a linear regression (R^2^=0.998) obtained from standard GABA solutions (40-200 mg/L) and their corresponding absorbance values.

### Measurement of γ-oryzanol mass fraction

For extraction of γ-oryzanol, 1 g of (germinated) brown rice flour was suspended with 4 and 8 mL of 2-propanol in two centrifuge tubes and vortexed for 1 min. After centrifugation (centrifuge 5810 R; Eppendorf AG) at 700×*g* and 30 °C for 10 min, 1 mL of the obtained supernatant was added to a quartz cuvette containing 3 mL isopropanol. The absorbance was measured at 326 nm using UV-Vis spectrophotometer Genesys™ 150 (Thermo Fisher Scientific). Mass concentrations of γ-oryzanol extracted with *V*(2-propanol)=4 (*γ*_1_) and 8 mL (*γ*_2_) were determined using a linear regression (R^2^=0.999) constructed from standard γ-oryzanol solutions (*γ*=25-125 mg/L) and their corresponding absorbance values. Finally, γ-oryzanol mass fraction in mg per gram sample was calculated according to the following equation (*1*, *14*):


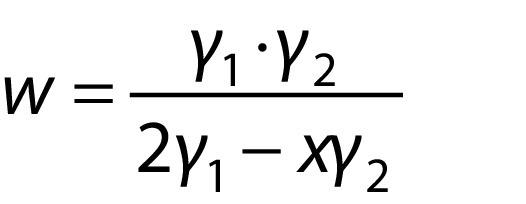


### Measurement of antioxidant capacity

The antioxidant capacity was measured by the change of colour of 1,1-diphenyl-2-picrylhydrazyl (DPPH) solution after the addition of germinated brown rice flour supernatant (*17*, *18*). The antioxidant activity of the sample was demonstrated by the change of the DPPH purple colour to the yellow colour of the final solution. Approx. 2 g of flour and 20 mL of 80% (*V*/*V*) ethanol were poured into a 50-mL centrifuge tube. The sample was extracted for 30 min at 30 °C, and shaken at 150 rpm (New Brunswick™ Innova® 2300; Eppendorf AG). The supernatant was obtained through centrifugation (centrifuge Z 366 K; Hermle-Labortechnik, Wehingen, Germany) at 4032×*g* and 4 °C for 30 min. Furthermore, 0.2 mL of the supernatant was mixed with 3.8 mL of 0.1 mM DPPH (in methanol). The mixture was incubated for 30 min in a dark room before analysis at a wavelength of 517 nm using UV-Vis spectrophotometer Genesys™ 150 (Thermo Fisher Scientific). Ascorbic acid was used to construct the linear regression (R^2^=0.996), and the antioxidant capacity was expressed in mg ascorbic acid (AA) per 100 g sample (*19*).

### Thermal properties of (germinated) brown rice flour

The analysis of the thermal properties of (germinated) brown rice flour followed Saif *et al*. (*20*), using a differential scanning calorimeter DSC-60 (Shimadzu, Kyoto, Japan). Approx. 8 mg of sample were poured into a specific aluminium DSC container and 16 μL distilled water were added. The container was closed and stored for 24 h to reach equilibrium. For measurement, the heating rate was set to 10 °C per minute with a scanning temperature range from 25 to 100 °C.

### Statistical analysis

The statistical analysis was performed using SPSS software v. 23 (*21*). The analysis of variance (ANOVA) and Duncan’s multiple range test were used to indicate significant differences among the tested samples (*22*). Within this study, three replicate experiments were performed for each soaking method. For GABA and γ-oryzanol mass fractions, and antioxidant capacity, each plotted value was a mean of nine measurements.

## RESULTS AND DISCUSSION

### pH profile during germinated brown rice production

**Fig. 2:**
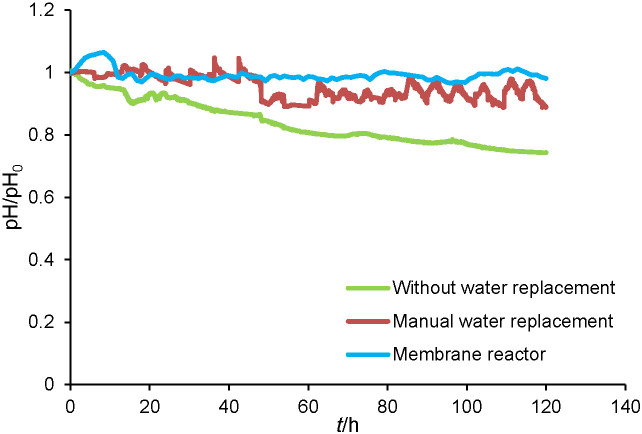
pH profiles of soaking water during the production of germinated brown rice with three soaking methods (*i.e.* without water replacement, with manual water replacement, and in a membrane reactor)

The pH profiles of soaking water from the three soaking methods (*i.e.* without water replacement, with manual water replacement, and in membrane reactor) had different trends (Fig. 2). These differences might be associated with the growth of microorganisms during those three soaking methods. The reduction of pH during soaking without clean water replacement was almost linear. Using the method where soaking water was manually replaced, the pH changed following the cycle of water replacement. As expected, the pH value of soaking water in the membrane reactor was maintained almost constant.

The near-linear reduction of pH (for up to 1.5) occurred during the soaking without water replacement. This trend was in line with Lu *et al*. (*23*), where pH was reduced from 6.6 to 4.1 within 24 h during the natural fermentation of raw milled rice. Kim *et al*. (*24*) found that the number of lactic acid bacteria (LAB) was approx. 1.8·10^5^ CFU/g in commercial germinated brown rice. The heterofermentative LAB might produce extracellular α‐amylase, which is responsible for starch hydrolysis. Moreover, LAB can metabolise the resulting reducing sugars to produce lactic (6.26 g/L) and acetic acid (0.36 g/L), leading to pH reduction (*23*). Besides, the continuous pH reduction did not occur during soaking with manual water replacement every six hours. For the first 48 h, pH was reduced throughout soaking and increased close to its initial pH value after intermittent water replacements. Herein, washing with regular clean water replacement almost completely removed lactic acid from rice grains. However, after 48 h of soaking, the pH was reduced significantly down to approx. 6.0. This decrease might be due to the excessive accumulation of lactic acid from the previous soaking period. Dzulfakar and Tan Kofli (*25*) also reported the same trend, where the second cycle of water replacement was not able to bring pH value close to the initial one. As mentioned above, the production of germinated brown rice using a membrane reactor could maintain the pH value of soaking water nearly constant. A stable pH value prevents LAB-based natural fermentation. The continuous washing followed by microfiltration could minimise the presence and the growth of LAB inside the reactor. Parallel to this, the introduction of continuous stirring eliminated the presence of dead zones (beneficial for LAB growth) due to the jumbled arrangement of brown rice inside the reactor. Persson *et al*. (*26*) also demonstrated the feasibility of microfiltration to separate lactic acid-producing bacteria from the fermentation broth.

### Physical appearance of germinated brown rice

**Fig. 3:**
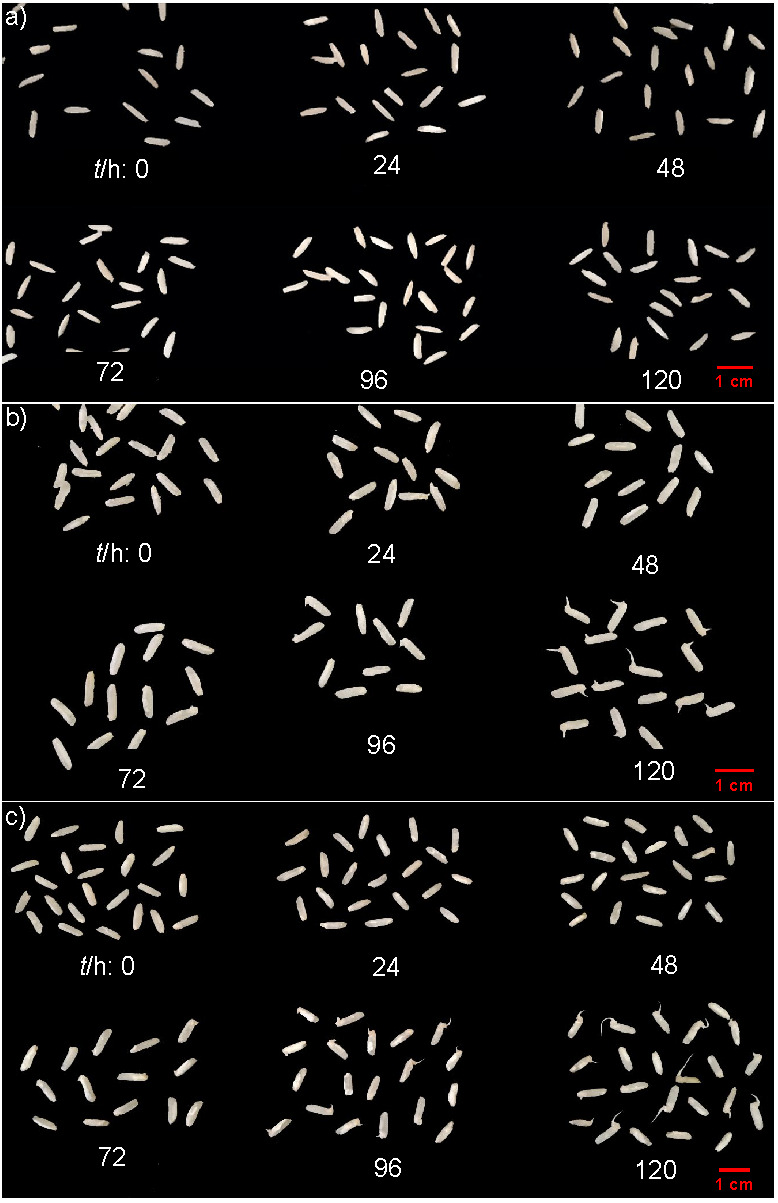
Morphologies of germinated brown rice produced using three different soaking methods: a) without water replacement, b) with manual water replacement, and c) in a membrane reactor

The morphologies of germinated brown rice produced with the three different soaking methods are presented in Fig. 3. Physical sprouts were almost not observable in germinated brown rice after soaking without water replacement at different soaking periods (0-120 h). The accumulation of lactic acid due to the natural fermentation of LAB, as shown in Fig. 2, was considered as an underlining factor to cause this. Thu Ha and Xuan (*27*) reported that lactic acid was detrimental for rice seed germination and shoot height. The addition of 0.5% lactic acid in soaking water stopped the germination of seven investigated rice varieties. As expected, the soaking with water replacement (manually every six hours or in the membrane reactor) could facilitate the germination of brown rice. However, the rice started to germinate earlier when soaked in a membrane reactor (72 h) than with manual water replacement (96 h). A small accumulation of lactic acid in the soaking with manual water replacement (see Fig. 2) might be attributed to this delayed germination. The length of the germinated brown rice sprouts in the membrane reactor was also higher than with manual water replacement. The continuous washing of the brown rice inside the membrane reactor was presumed to create an apt environment that could facilitate rice metabolism towards germination.

### Profile of GABA mass fraction during germination

The initial GABA mass fraction in brown rice variety IPB 3S was approx. 36.8 mg/100 g sample. The GABA mass fraction in Korean llpumbyeo variety is approx. 15.3 mg/100 g (*28*), in Taiwanese Tainung 71 (non-pigmented rice) 2.5 mg/100 mg and black glutinous (pigmented) rice 1.75 mg/100 mg (*29*), in Thai Pitsanulok-2, Chainat 1, Supan 1, Patum 1 and Kaw Dok Mali 105 it is 19, 24, 34, 39 and 51 mg/100 mg, respectively (*30*, *31*), and in Chinese 2011k3 rice cultivar 4.7 mg/100 mg (*32*). The mass fraction of GABA in rice seems to depend on the variety, pigmentation and location.

**Fig. 4:**
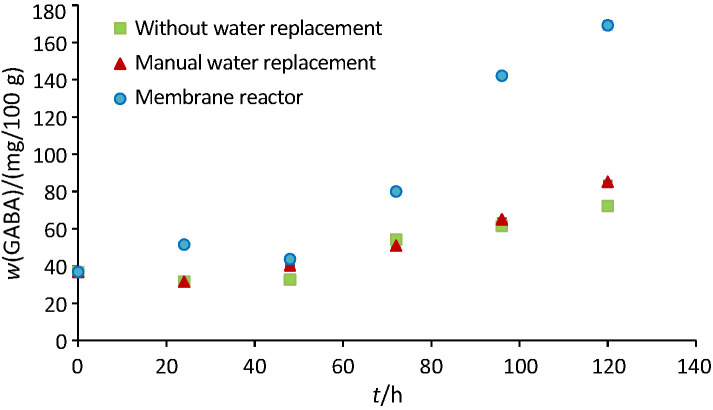
The effects of soaking methods on the γ-aminobutyric acid (GABA) mass fraction in germinated brown rice

As seen in Fig. 4, GABA mass fractions increased significantly during the three soaking methods (p<0.05). The GABA mass fractions after 120 h were 169.2, 85.3 and 72.2 mg/100 mg in germinated brown rice obtained in the membrane reactor, after soaking with manual water replacement and soaking without water replacement, respectively. Herein, the highest increase of GABA (more than 4.5-fold) was obtained in germinated brown rice produced in the membrane reactor, followed by the soaking method with manual water replacement (2.3-fold).

Several approaches reporting the increase of GABA mass fraction during rice germination in the past ten years are presented in Table 1 (*9*, *11*, *28*-*38*). Chen *et al*. (*9*) pretreated Chinese Taikeng 9 BR variety at a direct current (DC) voltage of 3 kV, with a constant current of 1.2 mA, and reduced pressure at 800 Pa for 10 min. GABA mass fraction increased 1.5-fold after germination. Plasma treatment increased the rate of water imbibition, which was considered vital for enhanced rice germination, thus increased GABA mass fraction (*9*). Zhang *et al*. (*11*) reported that by adding glutamic acid into the soaking water, GABA mass fraction in germinated brown rice increased. Besides increasing the GABA content, the addition of glutamic acid also shortened the germination time. As mentioned earlier, glutamic acid decarboxylase (GAD) in plants is responsible for synthesising GABA from glutamic acid and H^+^ ion. Therefore, the supplementation of glutamic acid in the soaking water positively increased GABA mass fraction in germinated brown rice. Cáceres *et al*. (*33*) reported the remarkable increase in GABA mass fraction during rice germination. By optimising the germination time and temperature, the enhancement of GABA mass fraction was 15- to 30-fold. The enhancement of GABA mass fraction in their study was also related to the activity of GAD at an optimised germination time and temperature.

**Table 1:** Current reports (past ten years) on the production of germinated brown rice with increased γ-aminobutyric acid (GABA) mass fraction due to different germination methods

Production condition	*w*(GABA)/(mg/100 g)	Enhancement/fold	Reference
*Oryza sativa* Japonica, Chinese Taikeng 9 (TK9) variety, pretreatment with a low-pressure plasma (direct current voltage of 3 kV and constant current of 1.2 mA for 10 min) followed by germination in Petri dishes at 25 °C for 24 h.	28.1	1.5	(*9*)
*Oryza sativa* Japonica, Chinese Jing 305 variety, soaked in water at pH=5.6 and 30 °C for 24 h with the addition of 1 g/L glutamic acid, followed by germination at 35 °C for 30 h with deionised water sprayed every 12 h.	140	-	(*11*)
*Oryza sativa* Indica, Chinese Guichao 2 variety	143	-	
*Oryza sativa* L., Korean Ilpumbyeo variety, completely soaked at 15 °C for 72 h with manual water replacement every 24 h.	26.84	2.2	(*28*)
*Oryza sativa* L., Taiwanese Tainung 71 variety (non-pigmented rice), soaked at 30 °C for 3 h followed by incubation on wet filter paper in a covered Petri dish at 35 °C and 100% relative humidity for 20 h.	6.23	2.5	(*29*)
Taiwanese black glutinous variety (pigmented rice)	4.88	2.8	
*Oryza sativa* L., Thai Pitsanulok 2 variety, soaked at 30 °C for 72 h with manual water replacement every 24 h, followed by incubation (germination) up to 30 days.	125	6.6	(*30*, *31*)
Thai Chainat 1 variety	159	6.7	
Thai Supan 1 variety	174	5.1	
Thai Patum 1 variety	201	4.8	
Thai Kaw Dok Mali variety	245	4.1	
*Oryza sativa* L., Chinese 2011k3 variety, presoaked in alkaline electrolysed water, followed by ultrasonication for 30 min and soaking in strong acidic electrolysed water (pH=3.27, oxidation-reduction potential=1024.10 mV and available chlorine concentration=17.76 mg/L) for 8 h, followed by germination on sterile cheese cloth at 25 °C and 85% relative humidity for 96 h.	24.36	5.2	(*32*)
*Oryza sativa* L., Ecuadorian INIAP 14 variety, soaked in water with pH=7, at 28 °C for 24 h, followed by incubation at 34 °C and 90% relative humidity for 96 h.	127.98	29.5	(*33*)
Ecuadorian INIAP 15 variety	139.32	29.7	
Ecuadorian INIAP 17 variety	129.47	25.5	
Ecuadorian GO39839 variety	123.92	15	
*Oryza sativa* L., Thai Niaw Dam Peuak Dam variety, soaked at (30±2) °C for 24 h, followed by steeped soaking with different buffers for 5 h, and finally germinated at (30±2) °C and 80-85% relative humidity for 24 h.	40.72	11.3	(*34*)
Thai Sangyod Phatthalung variety	44.53	16.7	
Thai Chiang Phatthalung variety	29.25	9.5	
*Oryza sativa* L., Thai RD-6 variety (waxy), soaked at room temperature for 12 h, followed by incubation in germinating cabinet for 24 h at 28-30 °C and 90-95% relative humidity.	68.4	2.4	(*35*)
*Oryza sativa* L., Chinese Dongnong 419, soaked for 12 h at 30 °C, followed by incubation for 24 h at 25 °C.	12.81	-	(*36*)
*Oryza sativa* L., Chinese Dongnong 419, segmented moisture conditioning in three stages with different contents of moisture (1.06, 1.42 and 1.31%), followed by incubation for 40 h at 25 °C.	28.14	-	
*Oryza sativa* L., Thai Kaw Dok Mali 105 variety, completely soaked at 25 °C for 72 h with manual water replacement every 4 h.	73.05	13.1	(*37*)
Thai Chainat 1 variety	92.42	12.8	
*Oryza sativa* L., Thai Kaw Dok Mali 105 variety, soaked with distilled water (pH=6) at 35 °C.	16.48	4.4	(*38*)
Thai Chainat 1 variety	14.50	4.5	
*Oryza sativa* L., Indonesia IPB 3S variety, soaked completely in water at pH=6.8±0.2 and (28±2) °C for 120 h with manual water replacement every 6 h.	85.29	2.3	This study
Indonesian IPB 3S variety, soaked completely in the membrane reactor with continuous washing at pH=6.8±0.2 and (28±2) °C for 120 h.	169.2	4.5	

The enhancement of GABA mass fraction was not as high as that of a study conducted by Cáceres *et al*. (*33*). Nevertheless, this study could mitigate the drawbacks of the conventional approaches (*i.e.* reducing wastewater and manpower). There was approx. 0.2 m^3^ wastewater discharged for producing 1 kg germinated brown rice with the manual soaking method (brown rice-to-water ratio of 1:10, soaking time of 120 h, water replacement every six hours). Soaking with a membrane reactor could reduce this amount down to one-sixth, approx. 0.033 m^3^. Furthermore, the membrane reactor merely needed the operator on duty only for the start and end activities. Germinated brown rice obtained in the membrane reactor had higher GABA mass fraction than the rice obtained by soaking with manual water replacement.

The occurrence of natural fermentation during germination with conventional methods (see Fig. 2) was presumably responsible for a small increase in GABA mass fraction. The microorganisms might actively degrade the sources of energy needed for rice germination (*i.e.* proteins, fats and carbohydrates), which are vital for the synthesis of RNA, protein and growth enzymes. Especially during soaking without water replacement, the domination of bacterial fermentation led to the smallest increase of GABA mass fraction after 120 h. Moreover, the production of germinated brown rice in the membrane reactor could circumvent the natural fermentation, which was beneficial for rice germination. Although the natural fermentation might inhibit germination, it also caused a small increase of GABA mass fraction in the germinated brown rice soaked without water replacement. Yokoyama *et al.* (*39*) reported the utilisation of *Lactobacillus brevis* IFO-12005 to synthesise GABA from glutamic acid in rice shochu distillery lees (*kome shochu kasu*). The presence of GAD is responsible for the ability of LAB to convert glutamic acid into GABA, and this synthesis starts from the late log phase to the stationary growth phase (*40*). The resulting GABA under this condition might penetrate the rice kernels as the rice texture is softened, leading to a small increase in GABA mass fraction. However, this presumption should be further verified.

### Profile of γ-oryzanol mass fraction during germination

**Fig. 5:**
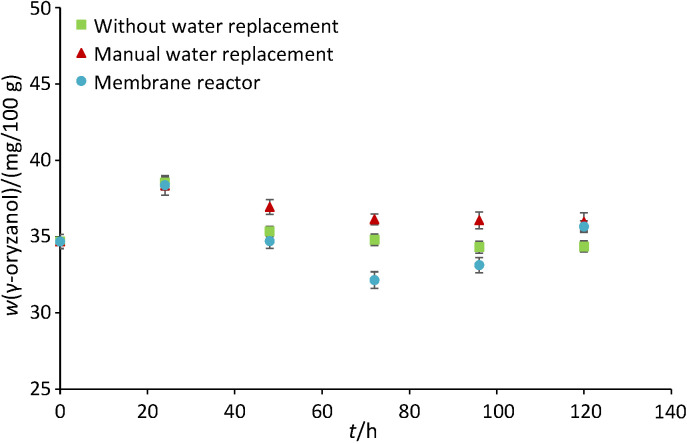
The effects of soaking methods on the γ-oryzanol mass fraction in germinated brown rice

The γ-oryzanol mass fractions in germinated brown rice produced with three different soaking methods had similar trends at various soaking times (Fig. 5). The γ-oryzanol mass fraction in brown rice was about 34.7 mg/100 g sample and increased similarly (*i.e.* up to 38 mg/100 g) during all three soaking methods after 24 h. After this soaking time, the mass fractions of γ-oryzanol reduced gradually and reached the lowest values at 72 h. The mass fractions started to increase again similarly during all three soaking methods until the end of germination at 120 h. Amongst three different soaking methods, germinated brown rice produced in the membrane reactor had the lowest γ-oryzanol mass fraction from 72 h onward. The γ-oryzanol is a non-polar fraction of the rice bran. It might be possible that γ-oryzanol was released into the soaking water, caused by the hydrodynamic shearing of the stirrers. Moreover, the mass fractions of γ-oryzanol were not significantly different at 72, 96 and 120 h (p>0.05) during soaking with and without manual water replacement. At 120 h, the γ-oryzanol mass fractions were 34.4, 36.9 and 35.7 mg/100 g in the germinated brown rice produced with the soaking without water replacement, with manual water replacement, and in the membrane reactor, respectively.

To our understanding, it is unclear whether the germination can increase or decrease γ-oryzanol mass fraction, as shown in our results. Kiing *et al*. (*41*) reported that rice cultivars of Udang Halus and Silah had the highest mass fraction of γ-oryzanol after 16 h of germination. After this, prolonged germination was detrimental for γ-oryzanol mass fraction. Moreover, other cultivars like Chelum, Sabak, Biris, Hitam, Boria and Mamut 2 showed different γ-oryzanol mass fractions within 24 h of germination. Ng *et al*. (*29*) reported fluctuations in γ-oryzanol mass fraction (48-65 mg/100 g) during Taiwanese black glutinous (pigmented) rice germinations (8-20 h). Banchuen *et al*. (*34*) obtained the identical γ-oryzanol mass fractions in brown rice and germinated brown rice in three Southern Thailand rice cultivars (Niaw Dam Peuak Dam, Sangyod Phatthalung and Chiang Phatthalung). In addition to this, Moongngarm and Saetung (*42*) also found there was no significant difference in γ-oryzanol mass fractions between non-germinated and germinated RD-6 rice varieties. In contrast to this, Cáceres *et al*. (*43*) reported up to a 17% reduction of γ-oryzanol content when brown rice of Indica SLF09 variety was germinated.

### Antioxidant capacity of germinated brown rice

**Fig. 6:**
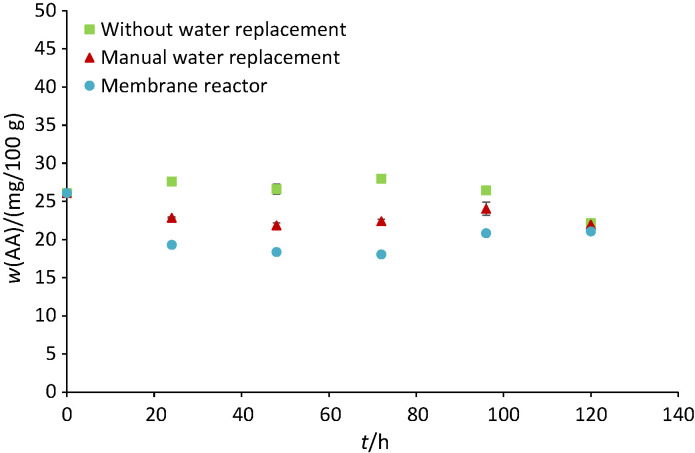
The effects of soaking methods on the antioxidant capacity expressed as ascorbic acid equivalents (AA) of germinated brown rice

The initial antioxidant capacity of IPB 3S variety expressed as ascorbic acid was 26.12 mg/100 g sample (Fig. 6). The antioxidant capacity of germinated brown rice produced without soaking water replacement increased in the first 72 h and was eventually reduced to 22.16 mg/100 g. The antioxidant capacity of germinated brown rice produced with manual water replacement varied, with a final antioxidant capacity at 120 h of 21.97 mg/100 g. The antioxidant capacity of germinated brown rice produced in the membrane reactor also decreased consistently in the first 72 h. However, a gradual increase was observed until the end of germination with a final antioxidant capacity of 21.07 mg/100 g. The antioxidant activities were significantly different (p<0.05) between the beginning and the end of soaking (a reduction trend). As for different soaking methods, the antioxidant activities were also significantly different (p<0.05), with the highest one obtained by soaking without water replacement.

The phenolic compounds are signalling compounds needed during germination (*44*). The activity of phenylalanine ammonia lyase (PAL), an enzyme that is responsible for synthesising phenolic acids, increases parallel to the increase of coleoptile size during germination (*45*). Therefore, germination could increase free and bound phenolics leading to the enhancement of antioxidant capacity (*10*, *45*). Besides having limited germination, increased antioxidant capacity was presumed due to natural fermentation of germinated brown rice without water replacement. LAB can synthesise feruloyl esterase (FE), which is capable of hydrolysing the ester bonds in plant cell walls. Ferulic acid and other hydroxycinnamic acids, which have high antioxidant activities, are then released by the action of this enzyme (*46*, *47*). However, as the lactic acid metabolism dominated, the activity of FE (optimum at pH=6.5 (*47*)) and germination rate reduced, causing a reduction of antioxidant capacity in germinated brown rice produced without water replacement. As mentioned above, phenolic acids located in the rice bran show higher antioxidant capacity than other compounds exhibiting antioxidant properties. The leaching phenomenon in repetitive soaking water replacement and hydrodynamic shearing caused by the agitation could reduce antioxidant capacity of germinated brown rice produced with manual water replacement and in the membrane reactor (especially in the first 72 h). Moreover, a gradual increase of antioxidant capacity in germinated brown rice produced in the membrane reactor indicated that germination prevailed over the leaching phenomenon.

### Thermal properties of germinated brown rice flour

**Fig. 7:**
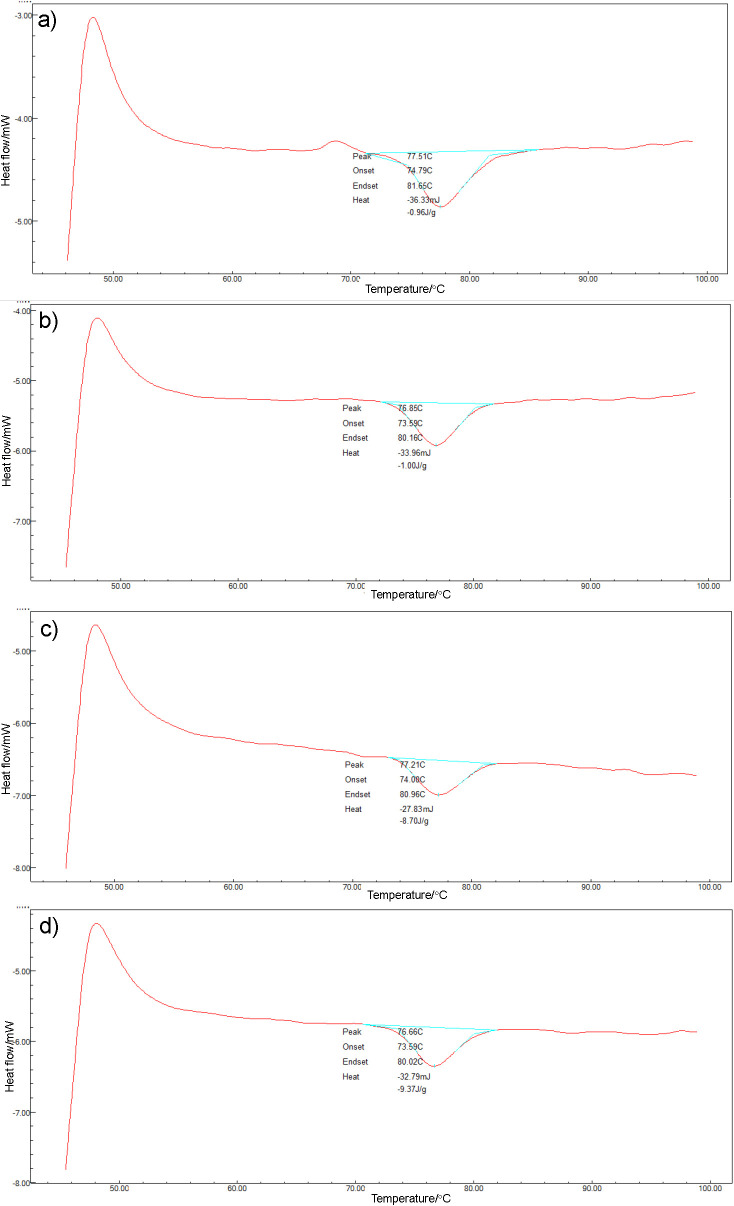
Differential scanning calorimetry thermograms of rice variety IPB 3S: a) brown rice, b) 120-hour germination without water replacement, c) 120-hour germination with water replacement every six hours, and d) 120-hour germination in membrane reactor

Within this study, germination also slightly reduced the transition temperatures of starch gelatinization of germinated brown rice (onset temperature, *t*_o_=73-74 °C, peak temperature *t*_p_=76-77 °C, and final temperature *t*_c_=~80 °C) (Fig. 7). Chung *et al*. (*48*) and Chinma *et al*. (*49*) reported similar trends for Korean Ilpumbyeo rice variety and three Nigerian rice varieties, respectively. According to Noda *et al*. (*50*) and Xu *et al*. (*51*), the increase of shorter amylose and outer chains in amylopectin caused lower *t*_o_ and *t*_p_ in sweet potato starch, buckwheat starch and brown rice flour. Moreover, Wunthunyarat *et al*. (*5*) also confirmed that during germination, by the actions of α-amylase, increased concentrations of saccharides with lower degrees of polymerization were observed.

## CONCLUSIONS

Within this study, the efficient production of germinated brown rice with increased γ-aminobutyric acid (GABA) mass fraction was demonstrated using a membrane reactor. The continuous washing of rice variety IPB 3S during germination enabled the circumvention of natural fermentation, which is favourable for brown rice germination. After 120 h of germination, GABA mass fraction increased 4.5-fold in the germinated brown rice produced in the membrane reactor, which was almost 2-fold higher than that of the soaking process with manual water replacement. The application of membrane reactor during germinated brown rice production also showed a substantial reduction in wastewater discharge and manpower on duty during the germination process. The impact of germination using a membrane reactor on γ-oryzanol mass fraction and antioxidant capacity was unclear. However, it is worth mentioning that one must carefully adjust the intensity of agitation during brown rice germination using a membrane reactor. Excessive hydrodynamic shear stresses exerted by the tips of the stirrer may leach out health-promoting components located in the rice bran (*e.g.* γ-oryzanol and phenolic compounds).
